# Determining Vaccination Frequency in Farmed Rainbow Trout Using *Vibrio anguillarum* O1 Specific Serum Antibody Measurements

**DOI:** 10.1371/journal.pone.0049672

**Published:** 2012-11-21

**Authors:** Lars Holten-Andersen, Inger Dalsgaard, Jørgen Nylén, Niels Lorenzen, Kurt Buchmann

**Affiliations:** 1 Department of Veterinary Disease Biology, Faculty of Health and Medical Sciences, University of Copenhagen, Frederiksberg C, Denmark; 2 Division of Veterinary Diagnostics and Research, National Veterinary Institute, Technical University of Denmark, Copenhagen, Denmark; 3 Aqua Denmark Unit, MSD Animal Health, Ballerup, Denmark; 4 Division of Poultry, Fish and Fur Animals, National Veterinary Institute, Technical University of Denmark, Aarhus N, Denmark; INRA, France

## Abstract

**Background:**

Despite vaccination with a commercial vaccine with a documented protective effect against *Vibrio anguillarum* O1 disease outbreaks caused by this bacterium have been registered among rainbow trout at Danish fish farms. The present study examined specific serum antibody levels as a valid marker for assessing vaccination status in a fish population. For this purpose a highly sensitive enzyme-linked immunosorbent assay (ELISA) was developed and used to evaluate sera from farmed rainbow trout vaccinated against *V. anguillarum* O1.

**Study Design:**

Immune sera from rainbow trout immunised with an experimental vaccine based on inactivated *V. anguillarum* O1 bacterin in Freund’s incomplete adjuvant were used for ELISA optimisation. Subsequently, sera from farmed rainbow trout vaccinated with a commercial vaccine against *V. anguillarum* were analysed with the ELISA. The measured serum antibody levels were compared with the vaccine status of the fish (vaccinated/unvaccinated) as evaluated through visual examination.

**Results:**

Repeated immunisation with the experimental vaccine lead to increasing levels of specific serum antibodies in the vaccinated rainbow trout. The farmed rainbow trout responded with high antibody levels to a single injection with the commercial vaccine. However, the diversity in responses was more pronounced in the farmed fish. Primary visual examinations for vaccine status in rainbow trout from the commercial farm revealed a large pool of unvaccinated specimens (vaccination failure rate = 20%) among the otherwise vaccinated fish. Through serum analyses using the ELISA in a blinded set-up it was possible to separate samples collected from the farmed rainbow trout into vaccinated and unvaccinated fish.

**Conclusions:**

Much attention has been devoted to development of new and more effective vaccines. Here we present a case from a Danish rainbow trout farm indicating that attention should also be directed to the vaccination procedure in order to secure high vaccination frequencies necessary for optimal protection with a reported effective vaccine.

## Introduction

Salmonid aquaculture has increased three-fold since 1980 and aquaculture in general is by far the fastest growing sector of food animal production in the world. In Denmark, where the focus is on rainbow trout, the production is equally expected to grow over the next few years. However, it is also expected that the increase in production should not lead to a corresponding growth in consumption of antimicrobials. Hence, the use of commercial vaccines against bacterial infections has spread and vaccination is now taking place at all farms with marine net cages as part of the production line. The commercial vaccine used in Denmark for injection vaccination is an oil-adjuvanted construct containing whole-cell antigen preparations of *Aeromonas salmonicida* subsp. *salmonicida* and *Vibrio anguillarum* serovar O1 and O2. However, despite the use of such vaccines infections with *V. anguillarum* might occur at the farms. This marine bacterium is found regularly on marine rainbow trout farms in Denmark and can be a significant cause of mortality in the stocked fish [1], [2]. While the level of protection from the vaccines may vary against *A. salmonicida* subsp. *salmonicida*, the vaccine efficacy against *V. anguillarum* is generally high [3]. Hence, we speculated that part of the explanation for the infections with *V. anguillarum* might be found in the vaccination procedure. To answer this question, we developed a highly sensitive and specific ELISA as a tool for monitoring vaccination frequencies in rainbow trout populations following large-scale vaccination procedures. With this assay we determined levels of anti-*V. anguillarum* antibodies in sera from farmed trout and compared these findings with visual examinations of the fish based on the Speilberg scale [4].

## Results

### Assay Characteristics

#### Coating antigen

**Figure 1:**
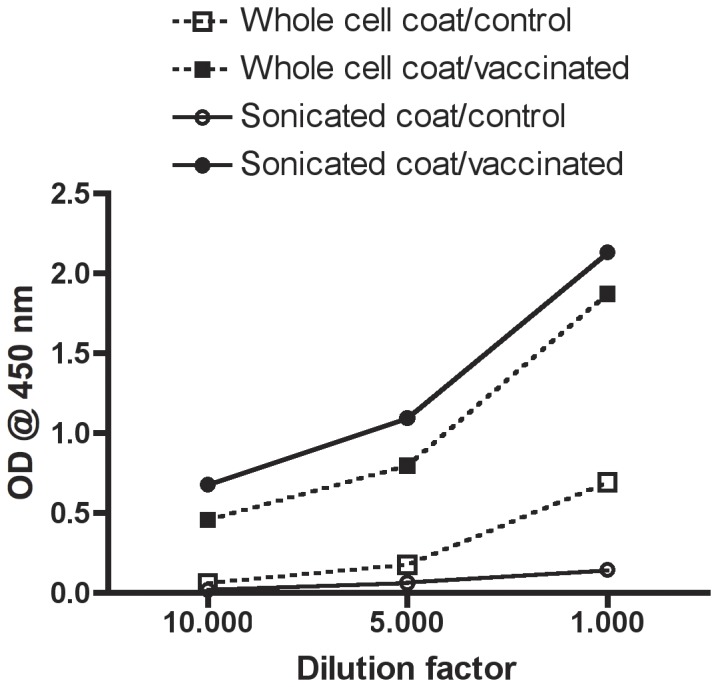
Performance of two different ELISAs coated with either whole cell or sonicated bacterial antigen. Serum pools from 20 unvaccinated (PBS injected, control) and 20 vaccinated (three immunisations) were analysed in 1∶1000, 1∶5000, and 1∶10000 dilutions.

Coating the microplates with sonicated bacteria compared with whole-cell antigen coating led to significantly different OD readings with serum (pooled, n = 20) from unvaccinated fish (control) diluted 1∶1000 (P<0.0001), 1∶5000 (P = 0.003) and 1∶10000 (P = 0.009) in assay buffer. OD values for unvaccinated fish were lowest in microplates coated with sonicated material (Fig. 1). The two coatings also produced significantly different readouts with serum (pooled, n = 20) from three times immunised rainbow trout (formalin-killed *V. anguillarum* O1 in FIA) diluted 1∶1000 (P = 0.012), 1∶5000 (P = 0.0004) and 1∶10000 (P = 0.0002). However, in the vaccinated fish the highest OD values were measured in microplates coated with sonicated bacteria. When antigen (*V. anguillarum* sonicate) was omitted from the coating buffer, reactions were not significantly different from blank wells (sample dilution buffer only; data not shown).

#### Dilution series and analytical signal

**Figure 2:**
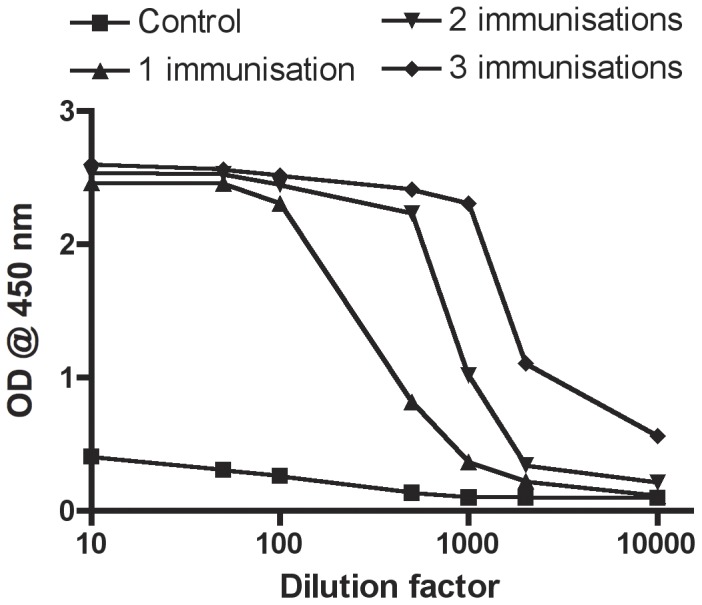
Titration curves based on pooled sera from 20 controls and 20 rainbow trout immunised i.p. one, two or three times with an experimental vaccine (formalin-killed *V. anguillarum* O1 in FIA).

Representative titration curves based on pooled sera from unvaccinated rainbow trout and sera pooled from fish immunised one, two or three times are shown in Fig. 2. The plotted data produced sigmoid curves for the immunised fish, whereas serum from the naïve fish generated a linear horizontal curve showing a slight increase in background at the highest serum concentrations. The absorbance at 450 nm against the serum dilution factor exhibited a linear relationship in a narrow dilution range for all three immune sera with slopes increasing with the number of immunisations. The limit of detection for the *V. anguillarum* O1 assay was an OD of 0.066.

#### Intra- and interassay precision

The analysis of eight determinations of the same serum sample on one microplate gave a CV≤3.4% at 1∶1000 and 1∶5000 dilutions. The interassay CV was ≤6.3% based on duplicates of both dilutions of the control serum pool on individual plates run on 14 different days.

*Stability* The level of specific antibodies in the serum samples remained unchanged through ten cycles of freeze/thaw. In addition, keeping samples at room temperature for 24 h had no effect on antibody levels (data not shown).

### Antibody Titre Increase for Each Immunisation

**Figure 3:**
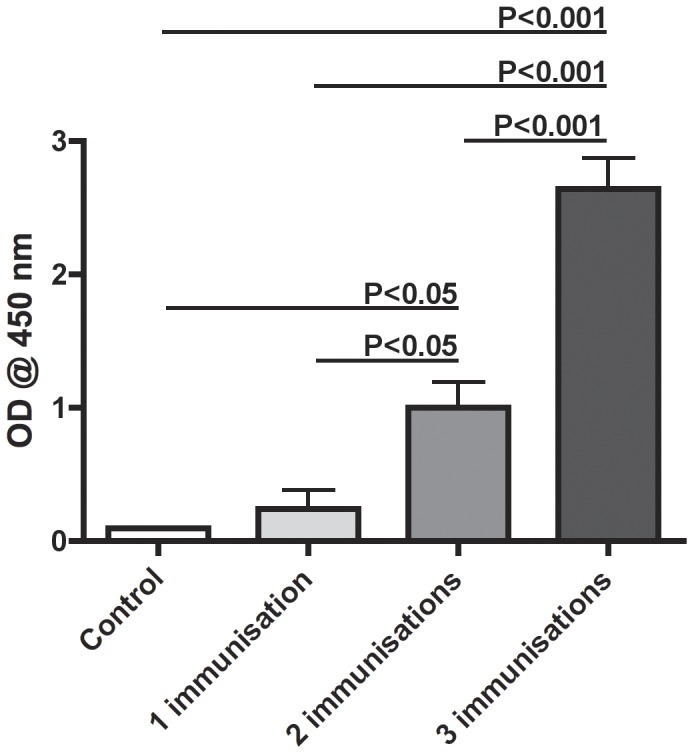
Antibody responses to repeated immunisations followed in 20 rainbow trout. Serum samples from 20 controls and 20 rainbow trout immunised i.p. one, two or three times with an experimental vaccine were analysed individually. All samples were diluted 1∶1000.

Sera from the fish that received three consecutive immunisations i.p. with a test vaccine were analysed individually for vaccine specific antibodies and Fig. 3 displays these data presented as OD values. As shown, there was no significant difference between control fish (0.10±0.0) and rainbow trout immunised once (0.24±0.14). However, following both the second (1.0±0.19) and third immunisation (2.64±0.23) a significant increase in *V. anguillarum* O1 specific antibodies was observed. Highest OD values were found with serum from rainbow trout immunised three times.

### Frequency of Unvaccinated Fish in a Population of Farmed Rainbow Trout

**Figure 4:**
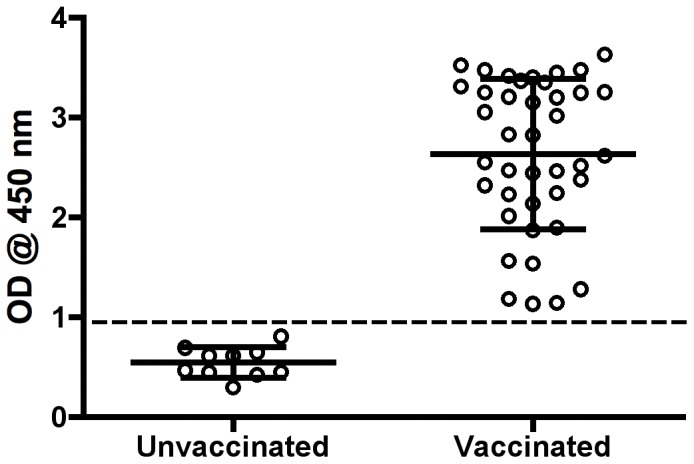
A scatter dot plot showing specific serum antibody responses from 50 farmed rainbow trout vaccinated once with a commercial vaccine against *V. anguillarum*. The dotted line represents the cut-off value for the ELISA at 0.97. All samples were diluted 1∶1000.

For a final evaluation of the *V. anguillarum* ELISA as a tool for determining vaccine status in farmed rainbow trout populations, blood samples were collected from 50 fish at a Danish sea farm. The fish were also examined for visual signs of previous vaccination. 15 months prior to this sampling 50 fish from the same batch of rainbow trout were randomly collected from the pond of supposedly vaccinated fish on the day of vaccination and visually examined for their vaccine status. The data from the visual examinations are presented in Table 1. There were found ten apparently unvaccinated fish (20%) out of the 50 examined specimens at both the fresh water and salt-water facility. This frequency of unvaccinated fish was confirmed with the ELISA (Fig. 4). OD values were significantly associated with the status as unvaccinated (P<0.0001) with an AUC of 1.00 (95% CI: 1.00–1.00). Moreover, the receiver operating characteristic (ROC) analysis showed 100% specificity and 100% sensitivity for the ELISA with an OD value cut-off set at 0.973. Based on these observations the 50 fish from the salt-water facility were divided into an unvaccinated and vaccinated group, and their data on size and condition are presented in Table 2. Although the unvaccinated fish were slightly longer compared to the vaccinated group (P = 0.041), the weight (P = 0.12) and average condition factor (P = 0.12) of the two groups were not significantly different.

**Table 1:** Descriptive data for 100 commercially farmed rainbow trout.

	N fish	Length (cm)	Weight (g)	Condition factor	Speilberg score	% unvaccinated
Fresh water, February 2009	50	19.5±1.5	83.4±19.6	1.12±0.10	–	20
Salt water, May 2010	50	38.7±3.8	763.1±204.7	1.29±0.12	1.25±0.54	20

Condition factor: CF = (W/L^3^)×100; where W = weight (g) and L = total length (cm) [18].

Speilberg score is a measure of vaccine-induced side effects.

**Table 2:** Descriptive data for 50 commercially farmed rainbow trout divided into unvaccinated and vaccinated groups.

	N fish	Length (cm)	Weight (g)	Condition factor
Unvaccinated	10	40.9±3.2	852.1±173.9	1.24±0.10
Vaccinated	40	38.2±3.8	740.9±207.7	1.30±0.12

Length: P = 0.041, weight: P = 0.12, condition factor: P = 0.12.

Condition factor: CF = (W/L^3^)×100; where W = weight (g) and L = total length (cm) [18].

## Discussion

Rainbow trout in culture suffer a variety of diseases, including bacterial, viral, parasitic and fungal infections. The majority of infections described are bacterial; including vibriosis caused by the extracellular bacterium *V. anguillarum*. Modern aquaculture takes prophylactic measures such as vaccination to combat serious infections, and with the continuous expansion of this industry there is an ongoing development of more effective ways to deliver vaccines to the fish [5]. In recent years refined machines for i.p. injections in fish have made automated vaccination a true alternative to manual vaccine delivery. However, the larger number of vaccinated fish per hour comes with an increased risk of vaccination failure if these machines are not handled optimally (personal observation). In Norway, being one of the largest producers of salmonids in aquaculture, the accepted vaccination failure rate at their fish farms is ≤2%. In Danish rainbow trout farming such a failure rate has yet to be agreed on.

The present study focused on the development and validation of an indirect ELISA for measuring anti-*V. anguillarum* O1 antibody levels as a marker for positive vaccination. The final assay was evaluated for its potential to determine the vaccination rate in a smaller population of rainbow trout subjected to automated vaccination against vibriosis at a Danish trout farm. Hence, the goal was to investigate and develop an assay useful for the farmers in determining the frequency of fish that accidentally have not received the vaccine after large-scale vaccination.

The development of a sensitive and specific ELISA requires special attention to a number of steps involved in the assay, here among the coating of the microplates. It has been described how optimising the coating process can lead to significantly better performance of the ELISA when the coating antigen is complex material like whole cell suspensions [6]. In our study, sonicated *V. anguillarum* O1 was found superior to whole cell *V. anguillarum* O1 as coating antigen in terms of significantly increased specific signal and lowered background. The reason for the better assay performance with sonicated coating material might simply be a larger number of exposed antigens on the bottom of the wells. Nonetheless, the result is a highly sensitive and specific ELISA, which is essential if the assay is to be used in screening for vaccination frequencies.

In the present study an experimental vaccine based on FIA and one inactivated bacterial isolate induced a weaker antibody response in rainbow trout compared to a commercial vaccine holding three bacterial isolates. However, the results were not directly comparable since fish stocks and rearing conditions differed. Moreover, there was a 15-month period between vaccination and serum sampling in case of the field-reared fish compared to 4½ months for the experimental fish. Nonetheless, the explanation for the observed difference could by a higher potency in multivalent vaccines [7]. Such an effect could possibly in part be attributed to a larger amount of bacterial immunomodulators in multivalent vaccines. Indeed, many of the most promising experimental adjuvant components are bacterially derived molecules that have been identified as highly conserved ligands for a range of immunologically important receptors on antigen presenting cells [8], [9]. Finally, it may not be excluded that cross-reactive antibodies targeted at such conserved and thus shared epitopes between *V. anguillarum* and *A. salmonicida* have been induced by the *A. salmonicida* component in the commercial vaccine.

High levels of anti-*V. anguillarum* O1 antibodies were measured with this ELISA in sera from sea-farmed rainbow trout. In agreement with a previous report our analyses revealed a large variation in specific antibody levels among truly vaccinated fish [10]. Since it was outside the scope of this study to investigate protective qualities of the antibodies measured with the described ELISA it is unresolved whether these differences in titres reflect different levels of protection against vibriosis. However, such specific antibodies have been shown to confer protection in rainbow trout against vibriosis [7], [11], [12]. Moreover, it has been suggested that the protective immune response in rainbow trout against extracellular bacteria, like in higher vertebrates, is predominantly humoral [13]. Hence, we speculate that high numbers of unvaccinated fish with a very low antibody response against *V. anguillarum* O1 as reported here could be part of the explanation for the vibriosis outbreaks observed at Danish sea farms [2]. However, further studies including larger populations of fish sampled at several farms are needed for conclusions on this theory.

There was not registered any outbreaks of vibriosis in the rainbow trout population during the study period. This might explain that the frequency of unvaccinated fish in the population had not decreased over time between the two sampling points. However, these outbreaks normally occur at the sea farms during summer when water temperatures are highest and hence would be expected to arise after the final sampling time point in the present study [1], [2]. Thus, it remains to be investigated how the presence and frequency of unvaccinated fish affects the risk and seriousness of an outbreak of vibriosis among the farmed fish. Possibly, herd immunity could be provided to a certain level by the 80% vaccinated fish. In terms of analysis of the antibody response in sea reared rainbow trout it also should be determined how an infection with *V. anguillarum* affects the antibody profiles of the fish. However, this will not hamper the usability of the ELISA for screening purposes since the testing of vaccination status normally will be performed before the fish are transferred to environments, e.g. sea cages, potentially infected with *V. anguillarum* O1. Indeed, the ELISA revealed that apparently unvaccinated fish, although low, still had an antibody response against *V. anguillarum* O1, which might be the result of natural exposure to environmental *V. anguillarum* after transfer to the sea cages. Optimally, an unvaccinated control group should have been included in the field study for interpretation of potential exposure to environmental *V. anguillarum*. However, this was not feasible since all fish intended for sea cage rearing in Denmark should be vaccinated. A maximum water temperature of 15°C monitored at the sea farm during the test period would explain the lack of disease outbreak despite potential bacterial exposure. Distinguishing between non-specific reactivity and low level specific reactivity is a key problem in the development of ELISA [14], [15]. To reduce the probability of false positive results (e.g. fish with detectable antibody levels induced by environmental bacterial exposure) it might be necessary, as in the present study, to set a relatively high cut-off value for the assay. However, this will also increase the probability of false negative results [10].

Apart from monitoring and improving efficacy of the vaccination procedure, knowledge on the immunological status of the actual fish population provided by the ELISA would help the farmer to handle vibriosis outbreaks in a more rational manner. As with other severe diseases vibriosis can lead to reduced net growth, increased need for treatment, and loss of fish dying from infection [16]. Thus, the ELISA presented here could be a useful tool for the fish farmer, and compared to visual examination it has the advantage that no fish needs to be killed in the process (sampling can be done on-site and the fish released).

## Materials and Methods

### Ethics Statement

The Committee for Animal Experimentation, Ministry of Justice, Copenhagen, Denmark, approved the study including the fish rearing and experimentation (license number 2006/561-1204), which was performed following the ethical guidelines listed in the license. All animal procedures were in agreement with the EU Directive 2010/63/EU for animal experiments?

### Fish and Rearing Conditions

Rainbow trout (Skinderup strain, Jutland, Denmark) were hatched and reared under pathogen-free conditions (Danish Centre for Wild Salmon, Randers, Denmark), before they were brought to our experimental fish keeping facility. The pathogen-free status of the fish was confirmed in the laboratory. 40 rainbow trout (average weight 25 g/fish) were split into two groups of 20 fish each. The fish were kept in 200 L tanks (20 fish/tank) with bio-filters (Eheim, Germany) and maintained at a 12 h light and 12 h dark cycle in aerated (100% oxygen saturation) tap water at 12°C. One group was injected once intraperitoneally (i.p.) with PBS (control) while the other group received three consecutive immunisations i.p. with an experimental vaccine.

A commercial aquaculture production line considered representative for Danish rainbow trout farming comprising both fresh water and salt-water facilities was selected for the field sampling. The rainbow trout population for this production line was vaccinated i.p. by machine at the fresh water farm in February 2009. The vaccine comprised inactivated *Aeromonas salmonicida*, *Vibrio anguillarum* serovar O1 and O2 emulsified in a non-mineral oil adjuvant (FuruVac 5 Vibrio; Intervet Schering-Plough Animal Health). On the day of vaccination 50 fish were randomly collected for visual examination just after passage through the vaccination machine. First, the fish were checked on the exterior for needle entries. Second, the abdominal cavity was opened with a scissor and both cavity and organs were thoroughly examined for presence of injected vaccine. In case of no vaccine in the abdomen the fish was checked for incorrect vaccination in the dorsal part. A year later, the rainbow trout population was transferred to sea cages. On one day in May 2010 50 trout were randomly taken from the cages. The individual fish length and weight was registered and blood samples collected. Finally, the 50 rainbow trout were dissected and thoroughly examined for visible signs of previous vaccination (fibrinous adhesions, pigmentation/melanin deposits and/or vaccine residues).

### Experimental Vaccine Preparation and Immunisation

48 h culture of the strain *V. anguillarum* O1 ATCC 43305 was inactivated with 0.9% formaldehyde for 2 h at room temperature, washed with PBS, adjusted to an optical density corresponding to 2×10^8^ cells/mL PBS, and emulsified with an equal volume of Freund’s incomplete adjuvant (FIA; Sigma, St. Louis, Mo.). Fish were immunised by i.p. injections with 0.1 mL of emulsified vaccine (1×10^7^ cells/injection). Immunisations were given three times with 500 day degree intervals (six weeks).

### Bacteria

The strain *Vibrio anguillarum* O1 6018/1 (ATCC 43305) was used for immunisations and coating of ELISA plates. Bacteria were grown with agitation for 48 h at 20°C in veal infusion broth with 1% NaCl and enumerated as colony forming units (CFU) on blood agar (blood agar base CM55, Oxoid, supplemented with 5% bovine blood). Stock cultures were maintained at −80°C in a broth culture supplemented with 15–20% glycerol.

### Serum

Blood was collected as individual samples by caudal venipuncture and serum obtained by allowing the blood to clot overnight at 5°C followed by centrifugation (10.000 rpm for ten minutes without brakes). Serum samples were stored at −80°C in aliquots of 0.5 mL. Time points for blood collections from laboratory fish were one week prior to each immunisation and six weeks after third and final immunisation.

Field samples were collected likewise. Directly after sampling, the blood was put on ice and kept cool until arrival at the laboratory five hours later. At the laboratory blood samples were placed at 5°C. Serum was isolated the following morning and stored at −80°C until use.

### ELISA for Determination of *V. anguillarum* O1 Specific Antibodies

For coating, each well of the microplates (flat-bottom 96-well plates, MaxiSorp™, Nunc) was filled with 100 µL coating buffer (C-3041, Sigma) containing 5 µg/mL of antigen (sonically disrupted *V. anguillarum* O1, Artek Sonic Dismembrator, Model 300, 60% for 3×5 min on ice; protein concentration determined with BCA Protein Assay, Thermo Scientific) and incubated overnight at 4°C. For comparison we also tested formalin inactivated whole-cell *V. anguillarum* O1 as coating antigen (5 µg/mL). After removal of coating solution, the wells were washed three times with 250 µL wash buffer (0.1% Tween 20 in PBS, pH 7.2) per well. For blocking residual protein binding capacity, PBS (200 µL) containing 10 g/L bovine serum albumin (BSA) was added to each well of the microplates, which were then incubated at room temperature for 1 h. After blocking, the microplates were washed three times, aspirated, sealed (microplate seal, Nunc) and stored at −20°C until use.

Serum samples were diluted in assay buffer (0.1% BSA and 0.1% Tween 20 in PBS, pH 7.2) and added to the microplates in duplicates with 50 µL per well. The microplates were sealed and placed at 4°C for overnight incubation. The next morning, the microplates were washed three times and 100 µL of a mouse anti-salmonid Ig antibody solution (MCA2182, AbD serotec, 1∶400 dilution in assay buffer) was added. After 1 h incubation at room temperature, the microplates were washed three times, and 100 µl were added to each well of a rabbit F(ab’)2 anti-mouse IgG:HRP solution (STAR13B, AbD serotech, 1∶400 dilution in assay buffer). Optimal concentrations of the commercial antibodies were established through chessboard titration experiments (data not shown). The microplates were left for additional 1 h incubation at room temperature followed by three washes. Substrate solution was added (100 µl/well of TMB, Sigma). After 10 min of incubation, stop solution (100 µl 1N HCL) was added, and the absorbance at 450 nm was measured with a PowerWave 340 (BioTek).

The limit of detection for the assay was determined as the concentration corresponding to the signal three standard deviations above the mean for blank wells (sample dilution buffer only). The linearity of signal as a function of dilution was investigated in pooled serum samples (n = 20) diluted in sample dilution buffer ranging from 1∶10–1∶10000. Intra-assay coefficient of variation (CV) was determined for eight duplicates of 1∶1000 and 1∶5000 dilutions of a control serum pool on the same assay plate. The inter-assay CV was determined for duplicates of 1∶1000 and 1∶5000 dilutions of the control serum pool on individual plates run on 14 different days. The stability of the antibodies in the serum samples was tested when exposed to repeated steps of freeze and thaw (samples frozen and thawed in the range one to ten times) or keeping the samples at room temperature for 24 h.

### Statistics

The Prism© software package (version 4.0 for Macintosh, GraphPad Software, Inc.) was used to manage data and for statistical analyses. Mean values and standard deviations were calculated and differences between means assessed by two-tailed unpaired t test. Sensitivity and specificity were summarised in a receiver operating characteristic (ROC) curve and area under the curve (AUC) given with 95% CI [17]. Moreover, the ROC analysis was used to identify an optimum cut-off value for the assay as the point giving the highest possible sensitivity (true-positive rate) in conjunction with the smallest false-positive fraction. The significance level was set at 0.05.
